# Taxonomic Variation in Coral Depletion at Orpheus Island (Inshore Great Barrier Reef, Australia) Linked to Unprecedented Rainfall and Hyposalinity

**DOI:** 10.3390/biology15090718

**Published:** 2026-05-01

**Authors:** Morgan S. Pratchett, Harrison Locke, Roemer Booij, Ewa Buczkowska, Raj H. Mathias, Jennifer Calcraft, Gideon Heller-Wagner, Scott F. Heron, Peter C. Doll, Mike J. McWilliam

**Affiliations:** 1 College of Science and Engineering, James Cook University, Townsville, QLD 4811, Australia; harrison.locke@my.jcu.edu.au (H.L.); roemer.booij@my.jcu.edu.au (R.B.); ewakarolina.buczkowska@my.jcu.edu.au (E.B.); rajheinz.mathias@my.jcu.edu.au (R.H.M.); scott.heron@jcu.edu.au (S.F.H.); peter.doll@jcu.edu.au (P.C.D.); mjmcwilliam@outlook.com (M.J.M.); 2 Orpheus Island Research Station, James Cook University, Townsville, QLD 4811, Australia; jenni.calcraft@jcu.edu.au (J.C.); gideon.hellerwagner@jcu.edu.au (G.H.-W.)

**Keywords:** coral reefs, disturbance, flooding, freshwater, mass mortality

## Abstract

Acute disturbances caused by changing environmental conditions are increasingly affecting the structure and function of coral reef ecosystems. Notably, changing rainfall patterns are leading to increasing incidence of hyposalinity. This study explored interannual changes in the overall cover and composition of hard corals (order Scleractinia) in Pioneer Bay, Orpheus Island, which was subject to hyposalinity during unprecedented high rainfall in February 2025. Hard coral cover declined 66.60%, from 41.66% (±1.22 SE) in September 2024 to 13.92% (±0.92 SE) in October 2025, with coral loss mostly apparent on the reef flat and reef crest. Coral loss was not equally apportioned among different coral taxa (genera), possibly reflecting differential susceptibility to hyposalinity based on specific physiology and habitat associations. The most pronounced declines were recorded among Merulinidae, which were the predominant hard corals on the reef flat in 2024. Soft corals were similarly affected and exhibited major declines in abundance on the reef flat. Hyposalinity is rarely considered among the suite of climatic disturbances that impact on coral assemblages and reef ecosystems, but this research shows that the potential ecological effects are very extensive, adding to the diversity of acute disturbances that will influence the structure coral reef ecosystems in the Anthropocene.

## Simple Summary

While the effects of increasing temperatures on reef-building corals are well known and widely documented, there is comparatively limited research showing how reef corals respond to periodic exposure to low salinity. Anthropogenic climate change is causing modified rainfall patterns, which will increase the likelihood of extreme rainfall events and flooding that can cause marked declines in local salinity. This study reveals extensive coral loss in shallow reef habitats in Pioneer Bay, at Orpheus Island on the inshore Great Barrier Reef, attributable to very low (hypo) salinity caused by very heavy rainfall in February 2025. We estimate that two-thirds of corals died as a consequence of this extreme climatic event, with near comprehensive coral mortality recorded in the shallowest parts of the reef. This study shows that extreme rainfall events have significant potential to affect near shore coral reef ecosystems, adding to the suite of environmental changes that may undermine the future condition, structure and function of coral reef ecosystems.

## 1. Introduction

Coral reefs and other coastal ecosystems are increasingly subject to acute disturbances that are caused or exacerbated by changing environmental conditions, such as severe marine heatwaves [1,2], de-oxygenation [3], and hyposalinity [4]. The foremost effect of changing environmental conditions on coral reefs is thermal bleaching [5,6,7], reflecting sustained and ongoing changes in temperature regimes and the sensitivity of corals to elevated temperatures. However, corals also have narrow salinity tolerances [8], and acute hyposaline conditions during extreme rainfall and/or freshwater flooding may cause extensive coral mortality [9,10,11].

The extent to which corals may be exposed to acute hyposaline conditions varies greatly among different reef habitats, with the lowest levels of salinity (≤10 ppt) often recorded in shallow and intertidal environments [12]. Potential exposure to hyposalinity conditions also depends upon the proximity to major catchments and rivers, which affect freshwater input, as well as reef aspect and habitat structure, which influence the mixing of freshwater and seawater [8]. The effects of hyposalinity on hard corals (order Scleractinia) and other sessile reef organisms depends upon the deviance in local salinity and the duration of such effects (i.e., dose–time response [8]). For the most part, hard corals can withstand short-term exposure (<12 h) to 50% reductions in ambient salinity (from 34–36 ppt down to 17–18 ppt), but prolonged exposure (<24 h) to these same levels often results in extensive coral mortality [13]. The minimum level of salinity that most corals can tolerate for ≥24 h is 15 ppt [14], though few experimental or field studies have considered salinity levels <20 ppt [13]. Even moderate reductions in local salinity may have significant physiological effects on hard corals, often leading to a reduced abundance of endosymbiotic dinoflagellates (family Symbiodiniaceae LaJeunesse et al. (2018)) and/or constraints in photosynthetic capacity [10], which can have lasting effects on coral condition and productivity [13]. Hyposalinity can also affect a broad range of other coral reef organisms, especially sessile and benthic organisms in shallow reef habitats [15,16].

Coral taxa vary widely in their susceptibility to acute environmental stress, reflected in differential mortality during major disturbances (e.g., cyclones and severe marine heatwaves), which is often based on their colony shape [17,18]. It is also apparent that some corals (e.g., *Stylophora pistillata* (Esper, 1792)) are disproportionately susceptible to hyposalinity [19], but the underlying basis of such taxonomic differences is unclear. It is largely expected that coral species that predominate in habitats and reef areas that are more exposed to hyposalinity will be pre-adapted and more resilient to periodic exposure to hyposaline conditions during periods of heavy rain and runoff [14,19].

The purpose of this study was to compare coral cover and composition across distinct reef zones at Orpheus Island (an inshore reef in the central Great Barrier Reef) before versus after acute hyposalinity stress caused by unprecedented rainfall in February 2025. Extensive coral sampling was conducted in October 2024 and September 2025, providing insights into changes in the abundance of predominant corals across different reef zones, reflective of localised coral mortality that might be attributable to acute hyposalinity. While there are multiple factors that may cause mortality or injuries to individual coral colonies, thereby contributing to interannual changes in coral cover [20,21], we would expect limited overall declines in hard coral cover in the absence of major acute disturbances [22]. Critically, there was limited evidence of significant heat stress recorded during the period of this study, which may be partly due to localised cooling caused by the low-pressure system (named 13U) that resulted in unprecedented rainfall (sensu [23]). Moreover, 13U crossed the coast south of Orpheus Island, such that there were likely to be limited destructive winds and waves produced by this system [24].

## 2. Materials and Methods

**Figure 1:**
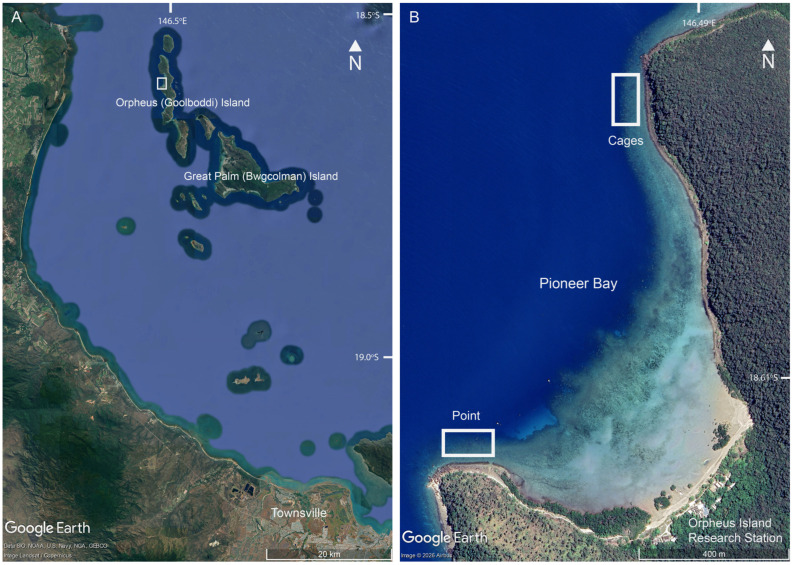
Satellite photographs from © Google Earth showing the study location, Pioneer Bay at Orpheus Island (**A**), and specific sites, Cages and Point within Pioneer Bay (**B**).

This study was conducted in Pioneer Bay, at Orpheus Island, which is a nearshore continental island located within the central region of Australia’s Great Barrier Reef (Figure 1). Pioneer Bay is located on the western (landward and generally sheltered) side of Orpheus Island, facing north-west. Pioneer Bay has a well-developed fringing reef with an extensive reef flat that largely comprises sandy habitat interspersed with patches of consolidated substratum [25]. The extent of consolidated substratum increases towards the outer edge of the reef flat, forming a continuous reef edge that slopes steeply to a sandy base at 6–8 m below mean sea level. The fringing reef is also much narrower at the northern and western extents of Pioneer Bay (Figure 1), where the consolidated substratum on the reef flat extends to the rocky (granite) shoreline. Sampling for this study was conducted within each of three distinct reef zones (reef flat, crest and slope) at each of two sites (Cages and Pioneer Point), located on the northern and western points of Pioneer Bay, respectively (Figure 1). The northernmost site is so named because it was the location of previous large-scaling caging experiments [26], though there is no evidence or legacy of this study.

### 2.1. Environmental Data

**Figure 2:**
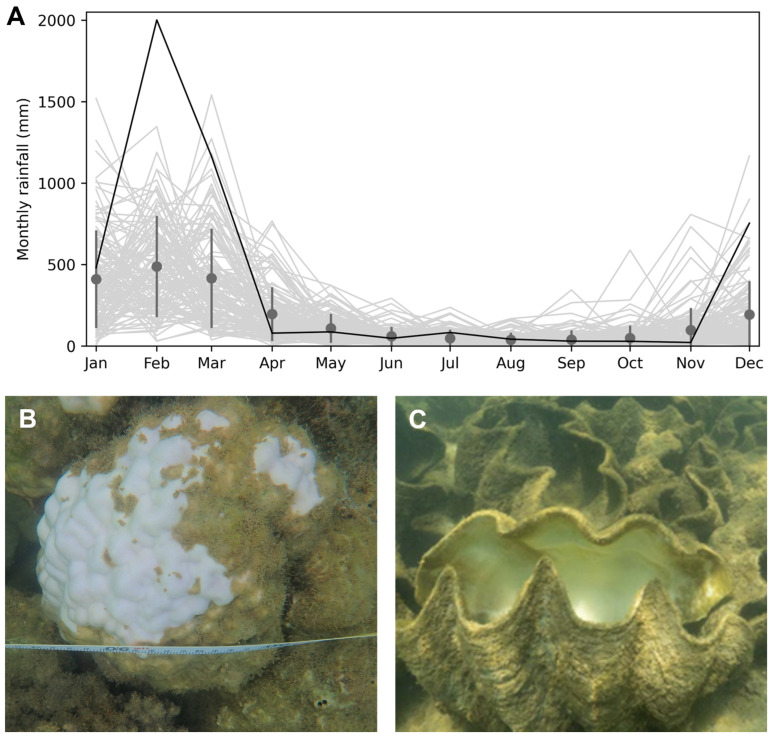
Total monthly rainfall (**A**) recorded at Halifax (Macrossan Street Station, 20 km west of Pioneer Bay) from June 1898 until December 2025. The dark grey line indicates the total monthly rainfall data recorded in 2025. The mean (and standard deviation) of the total monthly rainfall is shown for each month, calculated using the entire long-term data record. The apparent effects of hyposalinity on hard coral, massive *Porites* (**B**) and giant clams, *Tridacna gigas* Linnaeus, 1978 (**C**) in Pioneer Bay, in March 2025.

To establish the extreme and unprecedented nature of the rainfall event that occurred in early 2025, monthly total rainfall data was obtained from the Australian Government Bureau of Meteorology for the Halifax Macrossan Street Station, for which there is near complete (97%) monthly total rainfall data from June 1898 until December 2025. This is the closest weather station to Orpheus Island that has extensive and continuous rainfall data up until 2025. Monthly total rainfall was displayed for all years and also used to calculate mean and standard deviation of monthly total rainfall recorded since 1898 (Figure 2). The total monthly rainfall recorded in February 2025 was compared to the mean monthly rainfall for February since 1899, as well as the highest recorded monthly rainfall over the entire record.

Heat stress was assessed for 2024 and 2025 using the Degree Heating Week (DHW) produced by NOAA Coral Reef Watch. The DHW has been associated with moderate bleaching for values at or above 4 °C-weeks; and severe bleaching and moderate mortality above 8 °C-weeks [27].

### 2.2. Coral Sampling

To quantify benthic community composition, point intercept sampling was conducted along replicate 20 m transects deployed parallel to the depth contour within each zone and site. Benthic organisms and/or substrates underlying 40 uniformly spaced sampling points (50 cm apart) were recorded along each transect. Hard corals were identified to genus and further divided into distinct morphological categories, where relevant. All the students involved in collecting data were trained to distinguish predominant taxa using Coral Finder 2022 [28] with successive exposure to skeletal material, field images, and live specimens. Soft corals and abiotic components (rubble or sand) were also recorded. The predominant soft corals were *Lobophytum* Marenzeller, 1886 and *Sarcophyton* Lesson, 1834, but genera were not distinguished during surveys. The proportional cover of hard and soft corals was calculated relative to the extent of consolidated carbonate matrix and rubble, effectively accounting for areas of habitat that facilitate coral recruitment and growth [29]. A total of 562 transects were sampled across all zones, sites and years (Table 1).

### 2.3. Data Analyses

The proportional cover of hard corals (order Scleractinia Bourne, 1900) and soft corals (order Malacalcyonacea McFadden, van Ofwegen & Quattrini, 2022) were analysed using General Linear Mixed Models (glmmTMB package version 1.1.14 [30]) using a Tweedie distribution with a log link function in R version 4.5.1. [31] Both models tested for variation in coral cover (hard versus soft, respectively) between years (2024 and 2025), between sites (Point and Cages) and among zones (flat, crest and slope), with transect as a random effect. The DHARMa package version 0.4.7. [32] was used to assess model fit, confirming no significant deviations in residuals and predicted values.

Taxonomic variation in the responses of hard corals were analysed using MANOVA, testing for changes and shifts in coral composition (hard corals only) between years, sites and zones. To address taxonomic uncertainty [33] and also potential inconsistencies in taxonomic identification we merged taxa as appropriate to consider *Acropora* Oken, 1815; *Montipora* Blainville, 1830; *Pocillopora* Lamarck, 1816; branching *Porites* Link, 1807; massive *Porites*, Merulinidae Verrill, 1866; and Lobophylliidae Thiel, 1932, while all other hard corals (e.g., *Fungia* Lamarck, 1801 and *Turbinaria* Oken, 1815) were grouped into “other hard corals”.

### 2.4. AI Use

The graphical abstract was generated using Google Gemini (AI). The authors prompted the tool to show fringing coral reef with recent coral mortality on reef flat and reef crest, but high cover of live corals on the reef slope. Image was then further modified to show heavy rainfall and freshwater runoff concentrated through a creek that flooded on to the reef crest.

## 3. Results

### 3.1. Environmental Conditions

The total monthly rainfall recorded in February 2025 (2001.4 mm) was more than four times the average total February rainfall recorded since 1899 (488.2 mm). The highest monthly total rainfalls recorded prior to February 2025 were 1541.4 mm in March 1903, and 1346.9 mm in February 2009. While there were no consistent measurements of local salinity recorded in Pioneer Bay during the course of the flooding, opportunistic sampling that occurred in the aftermath of the peak rainfall event (on 8 February 2025) recorded very low salinity (10.0–11.7 ppt) in the surface water samples collected throughout Pioneer Bay (*n* = 6). It is likely that equivalent, or even lower, levels of salinity occurred over an extended period (from 4 to 12 February) coinciding with heavy rainfall and extensive runoff from the adjacent catchment on Orpheus Island. The highest tidal range that occurred during this period was 0.59–3.71 m (on 11 February 2025), and the reef crest and parts of the reef flat emerge at tidal heights of ≤0.60 m such that the corals in these habitats would have been directly exposed to very low salinity from rainfall and runoff, albeit for very short periods. Coastal flood waters originating from mainland catchments did not affect Pioneer Bay until after 17 February, and likely contributed very little to extreme hyposalinity.

During 2025, most heat stress accumulation occurred in January, with a peak of 4.33 °C-weeks in early February. At this level of heat stress, moderate coral bleaching would be expected, but little to no coral mortality [27,34].

### 3.2. Hard Coral Cover

**Figure 3:**
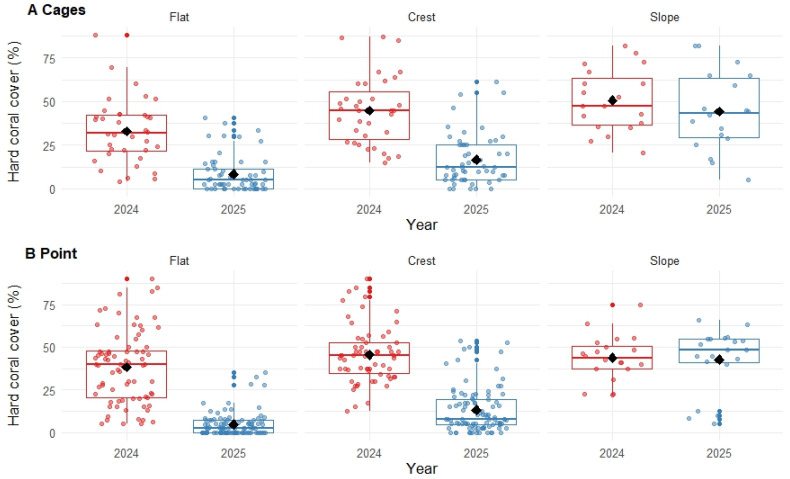
Box plots of hard coral cover for (**A**) Cages and (**B**) Point in Pioneer Bay, Orpheus Island (see Figure 1 for site positions), expressed as a percentage of available substrate. The black diamonds show the estimated means for each year and zone (flat, crest and slope), and the coloured dots show the distribution of data recorded on replicate transects (see Table 1 for the distribution of sampling effort). Interannual differences are significant for flat and crest at both sites, but not for slope at either site.

**Figure A1:**
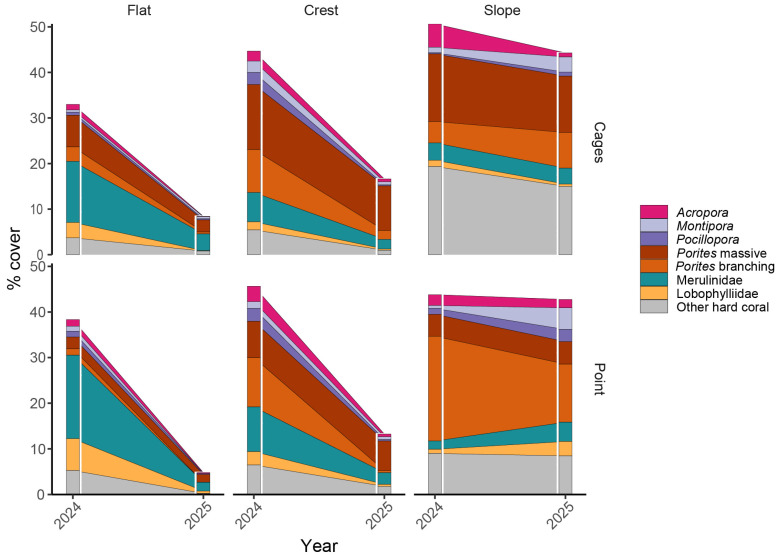
The change in cover (%) for each coral taxon, showing the cumulative changes in the hard coral cover at each site and zone, and the key taxa responsible for the documented changes (see also Figure 4).

The proportional cover of hard corals (order Scleractinia) declined 66.60% in Pioneer Bay, Orpheus Island, from 41.66% (±1.22 SE) in September 2024 to 13.92% (±0.92 SE) in October 2025. Declines in hard coral cover were highly pronounced on the reef flat and reef crest at both sites, whereas there was negligible change in coral cover recorded on the reef slope (Figure 3). The recorded declines in the mean cover of hard corals were 74.48% and 87.46% on the reef flat at Cages and Point, respectively. On the reef crest, declines were 62.80% at Cages and 70.99% at Point. On the reef slope, declines were 12.55% at Cages and 2.43% at Point. This shows that coral loss was generally higher at Point (versus Cages) on the reef flat and crest. Hard coral cover recorded on the reef slope at Point in 2025 was, however, highly bi-modal, where very limited coral cover (<15%) was recorded on seven (out of 17) transects, suggesting that there may have been some localised coral loss. The statistical model used to test for variation in hard coral cover (GLMM) revealed a significant interaction between year, site and zone (*p* = 0.02; Table A1), highlighting that interannual differences were not consistent across sites and zones. Site-level differences were somewhat negligible (Figure 3; Table A1), though levels of coral loss recorded at Point were higher than at Cages (Figure A1).

### 3.3. Soft Coral Cover

**Figure 4:**
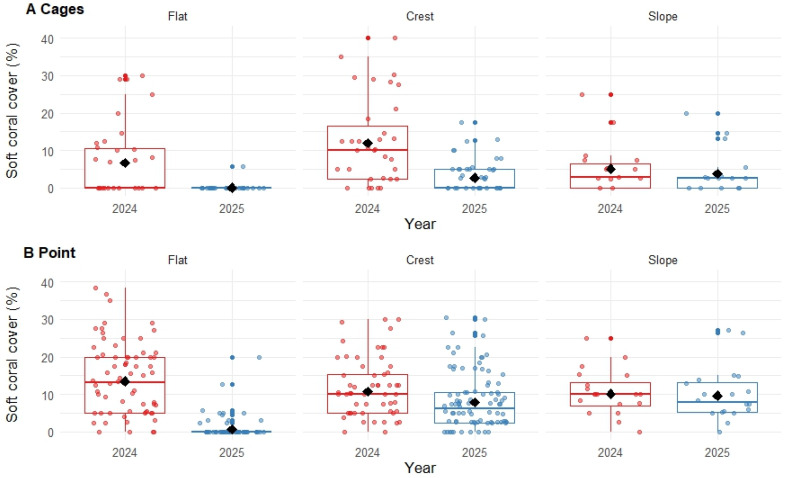
Box plots of soft coral cover for (**A**) Cages and (**B**) Point in Pioneer Bay, Orpheus Island (see Figure 1 for site positions), expressed as a percentage of available substrate. The black diamonds show the estimated means for each year and zone (flat, crest and slope), and the coloured dots show the distribution of data recorded on replicate transects (see Table 1 for the distribution of sampling effort). Interannual differences are significant for flat at both sites, and on the reef crest at Cages. There was no significant change in the cover of soft corals on the crest at Point, nor on the reef slope at either site.

The proportional cover of soft corals (order Malacalcyonacea) declined 68.14%, from 11.33% (±1.22 SE) in September 2024 to 3.61% (±0.92 SE) in October 2025. Declines in soft coral cover were particularly pronounced (>90%) on the reef flat, with comparatively limited declines recorded on the reef crest and slope (Figure 4). The statistical model used to test for variation in soft coral cover (GLMM) revealed a significant interaction between year and zone (*p* < 0.01; Table A2), emphasising that interannual differences varied among zones. There was also a significant interaction between zones and sites (*p* < 0.01; Table A2), whereby declines recorded on reef crest and slope were higher at Cages than at Point (Figure 4).

### 3.4. Taxonomic Variation in Coral Declines

**Figure 5:**
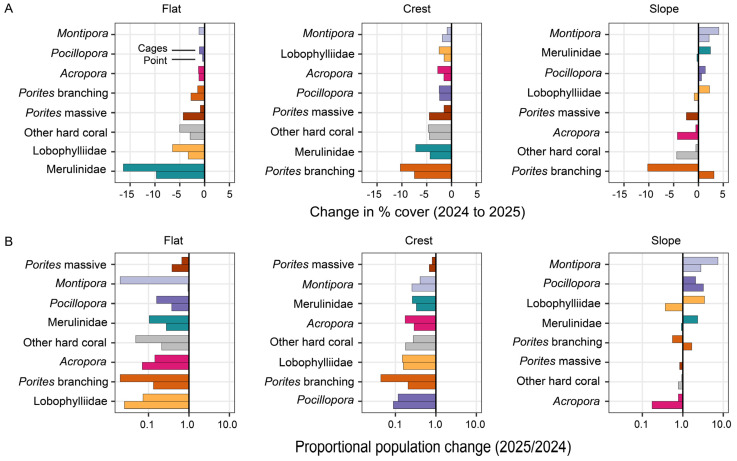
Change in abundance across coral taxa and relative susceptibility at each site and zone. (**A**) The absolute change in percent cover from 2024 to 2025. (**B**) A log-ratio plot highlighting relative susceptibility across populations, quantified as the ratio of the final (2025) and initial (2024) abundance on a log scale. In each panel, the changes at the two sites are shown (upper = Cages, lower = Point) and taxa are ranked from highest to lowest for each zone (ranks based on average changes at the two sites). Taxa are coloured consistently to facilitate contrasts among sites and zones.

Overall changes in hard coral cover recorded in Pioneer Bay from September 2024 to October 2025 were not equally apportioned across different coral taxa (Figure 5). The foremost contributors to documented declines in hard coral cover were the Merulinidae (including *Goniastrea* Milne Edwards & Haime, 1848 and *Coelastrea* Verrill, 1866), which were the predominant corals recorded on the reef flat in 2024 (Figure 5; see also Figure A1). However, relative declines in the cover of individual taxa were the greatest for branching *Porites* and Lobophyllidae which were scarcely recorded on the reef flat in 2025 (Figure 5). There were also large declines in the abundance of *Acropora*, *Montipora*, and *Pocillopora*, though these were rare on the reef flat even in 2024. On the reef crest, marked declines in cover of branching *Porites* contributed most to documented declines in hard coral cover. However, there were large relative declines in several less abundant taxa, including *Pocillopora* and *Acropora*. The cover of *Acropora* spp. also declined on the reef slope at Point, whereas there was limited change in other taxa on the reef slope (Figure 4). The statistical model used to test for changes in coral composition (MANOVA) revealed a significant interaction between year, site and zone (Table A3), highlighting that interannual differences were not consistent across sites and zones.

## 4. Discussion

Recent and substantial declines in hard coral cover in Pioneer Bay, which were most pronounced on the reef flat and crest, are most likely caused by unprecedented rainfall and extreme hyposaline conditions that occurred in February 2025. The total monthly rainfall recorded in February 2025 in the vicinity of Orpheus Island (>1200 mm) was more than four times the February average recorded since 1899 (Figure 2). More specifically, the February 2025 rainfall exceeded the February monthly average by nearly five standard deviations (indicating a likelihood of 1 in nearly 3.5 million). The depth-stratified pattern of coral loss is also consistent with expected patterns of coral mortality caused by significant freshwater input and associated hyposalinity [13]. Moreover, many of the corals in Pioneer Bay were observed to be bleached and there was extensive mortality recorded among other non-coral invertebrates (especially giant clams, *Tridacna gigas*) immediately following the peak rainfall and runoff (Figure 2).

The extreme levels of hyposalinity (≤10 ppt) recorded in Pioneer Bay likely persisted for several days, which greatly exceeds reported coral tolerances [9,10,13,35] and would be expected to cause substantial mortality among hard and soft corals. It is also expected that hyposalinity would have attenuated with increasing depth, thereby explaining the limited coral mortality recorded on the reef slope, and ameliorated at locations with more rapid and consistent mixing of freshwater with sea water [35]. Exposure to very low hyposalinity was likely moderated periodically by high tides, where corals on the reef flat may have been directly exposed to freshwater at low tides, which were as low as 0.59 m. It is unknown to what extent corals in other bays or reefs were impacted by this hyposalinity event, but it is possible that prolonged exposure to extreme hyposalinity was somewhat localised, both due to the shape and orientation of Pioneer Bay, but also the extensive runoff from the adjacent catchment that created a temporary river on the southern side of the research station in Pioneer Bay.

There are many reports of localised coral mortality and other sessile reef organisms resulting from hyposalinity caused by heavy or prolonged rainfall and runoff [13]. In 1998, in Kaneohe Bay, Hawaiʻi, comprehensive coral mortality occurred across a broad range of shallow reef habitats following low (≤20 ppt) salinity in surface waters that persisted for at least 4 days [36]. Hyposalinity also has a pervasive influence on the distribution and formation of near shore reefs [13,35], where coral growth and reef formation are highly constrained in areas that are periodically exposed to hyposalinity. Given the extent of fringing reefs in Pioneer Bay, and the reported levels of coral cover and diversity in previous years [22], it does appear that the acute hyposalinity stress that occurred in 2025 is a rare event, if not unprecedented. However, this latest disturbance adds to a series of major acute disturbances that have significantly affected coral assemblages in this region [22].

Substantial declines in the cover of both hard and soft corals were recorded in Pioneer Bay, suggesting that there was limited taxonomic variation in the capacity to withstand the acute hyposalinity to which the corals were exposed, especially on the reef flat and crest. Previous studies have suggested that massive coral species (e.g., Merulinidae and Lobophyllidae) are generally more resilient to hyposalinity [35,37], thereby explaining their predominance in areas (including shallow and intertidal habitats) that are regularly exposed to reduced salinity. This study suggests that even these massive corals have a limited capacity to withstand extreme or prolonged exposure to hyposalinity, while high absolute levels of coral mortality reflect their predominance in habitats that were most exposed to hyposalinity (Figure 5). This disturbance also further supressed the local abundance of branching corals (*Acropora* and *Pocillopora* sp.) in Pioneer Bay [22], which are important contributors to habitat structure [38] and also very important in facilitating coral recovery [39,40]. These corals are disproportionately susceptible to a range of different disturbances, including hyposalinity [17,37,41], but nonetheless dominate in many shallow reef environments [36] due to their rapid growth and recovery capacity [40]. The limited abundance of *Acropora* and *Pocillopora* spp. in Pioneer Bay suggests that major disturbances are occurring too frequently to allow for recovery [42], and the recovery of other slow-growing coral species (e.g., massive *Porites*) is likely to be extremely protracted in these habitats.

Although hyposalinity is likely the foremost cause of the local coral mortality that occurred in Pioneer Bay during the unprecedented rainfall event, the high levels of sedimentation and eutrophication that typically occur during extreme rainfall and flooding [11] may have exacerbated metabolic stress among zooxanthellate organisms [43]. Most critically, extensive runoff from the Queensland coast may have amplified and extended the environmental stress caused by acute hyposalinity from localised rainfall and runoff. Similarly, whilst the level of heat stress was unlikely to have resulted in the observed coral mortality, it may have caused some bleaching and therein exacerbated the effects of hyposalinity. This study does not have the necessary temporal resolution to assess the independent contributions of these different environmental stressors, though it is clear that there were substantial and immediate effects from the acute hyposalinity (Figure 2), which likely caused the ultimate mortality of many corals.

## 5. Conclusions

The inherent variability in environmental conditions means that coral reef ecosystems will be naturally subject to extreme conditions over multi-decadal time frames [36]. These rare events can have catastrophic short-term impacts, but also impose significant influence on the distribution and structure of coral reef organisms and habitats [13,35]. While hyposalinity is rarely considered among the suite of climatic disturbances that will increasingly impact coral assemblages and reef ecosystems, there are observed and projected changes in the intensity of rainfall events due to anthropogenic climate change [44]. Projections of increased heavy rainfall events also have a high degree of confidence [45]. Hyposalinity is therefore likely to have significant impacts on coral assemblages. Most importantly, acute exposure to hyposalinity will compound upon other escalating climate impacts, especially heat stress events, affecting the structure and function of coral assemblages and potentially undermining opportunities for natural adaptation.

## Figures and Tables

**Table 1:** Distribution of sampling effort (number of replicate transects) in each zone (flat, crest and slope) and site (Cages versus Point) for each sampling period. There were consistently fewer transects sampled on the reef slope owing to the need for SCUBA to effectively sample within deeper habitats. The depth is the mean water depth recorded over the substrate for each transect during actual sampling, which reflects the variation in tidal height whereas the depth of each zone relative to the mean sea level is quite consistent.

	Site 1 (Cages)			Site 2 (Point)		
Zone	Flat	Crest	Slope	Flat	Crest	Slope
Depth	2.35 m	3.13 m	5.41 m	1.30 m	2.16 m	5.63 m
2024	37	37	19	76	62	20
2025	53	51	16	90	84	17

## Data Availability

The raw data will be made available by the authors upon request.

## Appendix A

**Table A1:** Analysis of deviance (using Type III Wald chi-square test) for hard coral cover, showing statistical significance of factors (year: 2025 and 2025; site: Cages and Point; zone: flat, crest, and slope) and their interactions, fitted using a GLMM (Tweedie distribution with a log link function) with transect as a random effect. Bold rows signify *p* < 0.05.

Factor	χ^2^	d.f.	*p*
**Year**	**57.71**	**1**	**<0.01**
Site	0.04	1	0.83
**Zone**	**10.30**	**2**	**<0.01**
Year × Site	2.25	1	0.13
**Year × Zone**	**32.41**	**2**	**<0.01**
Site × Zone	2.44	2	0.29
**Year × Site × Zone**	**7.44**	**2**	**0.02**

**Table A2:** Analysis of deviance (using Type III Wald chi-square test) for soft coral cover, showing statistical significance of factors (year: 2025 and 2025; site: Cages and Point; zone: flat, crest, and slope) and interactions, fitted using a GLMM (Tweedie distribution with a log link function) with transect as a random effect. Bold rows signify *p* < 0.05.

Factor	χ^2^	d.f.	*p*
**Year**	**32.53**	**1**	**<0.01**
Site	0.09	1	0.77
**Zone**	**8.40**	**2**	**0.01**
**Year × Site**	**13.94**	**1**	**<0.01**
**Year × Zone**	**20.95**	**2**	**<0.01**
**Site × Zone**	**8.22**	**2**	**0.01**
Year × Site × Zone	2.49	2	0.29

**Table A3:** Multivariate analysis (using Piallai’s Trace) of coral composition, based on coral genera and morphotypes (hard corals only) showing statistical significance of factors (year: 2025 and 2025; site: Cages and Point; zone: flat, crest, and slope) and interactions. Bold rows signify *p* < 0.05.

Factor	Pillai	d.f.	*p*
**Year**	**0.52**	**1**	**<0.01**
**Site**	**0.13**	**1**	**<0.01**
**Zone**	**0.57**	**2**	**<0.01**
**Year × Site**	**0.69**	**1**	**<0.01**
**Year × Zone**	**0.32**	**2**	**<0.01**
**Site × Zone**	**0.18**	**2**	**<0.01**
**Year × Site × Zone**	**0.10**	**2**	**<0.01**
